# Targeting Integrin α2 to Overcome Imatinib Resistance in Chronic Myeloid Leukemia Cells

**DOI:** 10.3390/biom15091245

**Published:** 2025-08-28

**Authors:** Yalda Hekmatshoar, Tulin Ozkan, Arzu Zeynep Karabay, Sureyya Bozkurt, Aynur Karadag Gurel, Ozlem Kurnaz Gomleksiz, Tunc Fisgin, Asuman Sunguroglu

**Affiliations:** 1 Department of Medical Biology, School of Medicine, Altinbas University, 34147 Istanbul, Turkey; ozlem.kurnaz@altinbas.edu.tr; 2 Department of Medical Biology, School of Medicine, Ankara University, 06230 Ankara, Turkey; asuman.sunguroglu@medicine.ankara.edu.tr; 3 Department of Biochemistry, Faculty of Pharmacy, Ankara University, 06560 Ankara, Turkey; akarabay@ankara.edu.tr; 4 Department of Medical Biology, School of Medicine, Istinye University, 34396 Istanbul, Turkey; sbozkurt@istinye.edu.tr; 5 Department of Medical Biology, Gulhane Faculty of Medicine, Health Sciences University, 06018 Ankara, Turkey; aynur.karadag@sbu.edu.tr; 6 Central Research Laboratory, Altinbas University, 34147 Istanbul, Turkey; 7 Department of Pediatric Hematology-Oncology and Transplantation Unit, School of Medicine, Altinbas University, 34147 Istanbul, Turkey; tunc.fisgin@altinbas.edu.tr

**Keywords:** chronic myeloid leukemia, imatinib resistance, integrin α2, apoptosis, E7820

## Abstract

Chronic myeloid leukemia (CML) is a blood disorder caused by a genetic alteration that creates the *BCR-ABL* fusion gene, leading to continuous activation of cell growth signals and uncontrolled proliferation of the blood cells. Imatinib (IMA) resistance remains a major obstacle in CML treatment. Integrins, particularly integrin α2 (*ITGA2*), have been associated with cancer progression and drug resistance. In the current study, we investigated the role of *ITGA2* in IMA resistance using IMA-sensitive K562 (K562S) and IMA-resistant K562 (K562R) cells. Our findings showed that *ITGA2* is overexpressed in K562R cells and ITGA2 inhibitor E7820 (2.5 µM) treatment significantly decreased cell viability and induced apoptosis in both sensitive and resistant cells. Combination treatment with E7820 and imatinib enhanced pro-apoptotic gene expression (*BAX*, *BIM*) and decreased anti-apoptotic *BCL2* levels in imatinib-resistant K562R cells. Flow cytometry confirmed ITGA2 inhibition at the protein level, and rhodamine assays revealed reduced MDR1 activity in treated cells. These results demonstrate that targeting *ITGA2* may overcome imatinib resistance and offer a novel therapeutic strategy for CML.

## 1. Introduction

Chronic myeloid leukemia (CML), a type of blood disorder, arises from a reciprocal translocation between chromosomes 9 and 22 [t(9;22)(q34;q11)], causing the formation of the *BCR-ABL1* fusion gen [1]. BCR-ABL is a tyrosine kinase which is constantly active in CML [2]. CML can be effectively treated by tyrosine kinase inhibitors (TKIs), including imatinib (IMA), dasatinib, nilotinib, and bosutinib [3]. Additionally, about 10% of patients treated with IMA have mutations in the *BCR-ABL* fusion gene, leading to therapeutic resistance, and about 16% of patients develop an intolerance against TKIs [4]. The development of resistance to IMA is a major factor responsible for failure in therapy. There are different BCR-ABL independent mechanisms which are involved in IMA resistance, including overexpression of efflux proteins, resistance to apoptosis induction, DNA repair, and metabolic adaptation [5,6]. Recent studies represented the role of cell-extracellular matrix (ECM) or cell-cell adhesion in apoptosis and cell survival regulation. Moreover, it is also contributed to development of chemotherapeutic resistance in different cancers [7,8]. Leukemia cells have been shown to develop chemo-resistance via direct interaction with bone marrow (BM) stromal cells, a phenomenon known as cell adhesion-mediated drug resistance [7].

The integrin family comprises of heterodimeric cell adhesion receptors that play significant roles in the regulation of cell growth and function [9]. Each receptor is composed of one alpha (α) and one beta (β) subunit [8]. In vertebrates, integrins form 24 distinct heterodimers, each consisting of 1 of 18 alpha subunits paired with 1 of 8 beta subunits [10].

Integrin adhesion to stromal cells or ECM constituents in hematopoietic and epithelial cells triggers signaling necessary for cell migration, survival, and proliferation [8]. The investigation of new therapies targeting various integrins implicated in a diverse range of diseases is a rapidly advancing area in both pre-clinical and clinical research [9,11]. In both normal hematopoiesis and leukemia, the BM microenvironment plays a central role in regulating the homing, survival, and egression of cells, with integrins playing a crucial part in these processes [12].

Integrin α2 subunit (CD49b, encoded by the gene *ITGA2*) expression can differ significantly among healthy individuals, and this variability is partly due to single nucleotide polymorphisms (SNPs), which may influence transcription rates by modifying the binding affinity of transcription factors [13]. It binds only to β1 subunits and has a broad distribution in fibroblasts, endothelial cells, epithelial cells, and blood cells [14]. The α2β1 integrin is found in various cell types; however, within the hematopoietic lineage, its expression is limited to megakaryocytes and platelets [15]. Integrin α2β1-mediated collagen binding supports the survival of malignant T cells in acute lymphoblastic leukemia (ALL) [16].

*ITGA2* gene expression was found higher in 134 de novo acute myeloid leukemia (AML) patients in comparison with the 33 controls [17]. Furthermore, integrin α2β1 has been linked to the promotion of metastasis in several cancer types. In mouse models of breast, melanoma, and colon cancer, inhibiting integrin α2 was shown to reduce metastatic spread to the liver [18].

In our previous study, we reported that imatinib-resistant cells (K562R) represented stem cell characteristics and high expression of cell surface markers including integrins specially *ITGA2* compared to the imatinib-sensitive (K562S) cells [6]. Therefore, we chose to investigate the role of *ITGA2* in imatinib resistance in CML based on this finding. In the current study, we aimed to clarify the role of integrin-mediated imatinib resistance in CML by inhibiting ITGA2 using E7820 and analyzing its effects on cell proliferation and apoptosis pathway induction in CML cells.

## 2. Materials and Methods

### 2.1. Cell Culture

K562S (parental) and K562R cells were maintained in RPMI 1640 medium containing 10% fetal bovine serum (FBS) (Sigma, St. Louis, MA, USA). The 0.6 µM imatinib-resistant K562R cell line was kindly provided by Prof. Carlo Gambacorti-Passerini. Later, the 5 µM resistant K562R cell line was produced by gradually increasing the IMA (Santa Cruz, Dallas, TX, USA) concentration over a period of 18 months using the K562R 0.6 cells as the starting population [5]. For experimental purposes, K562R cells were further divided into two subgroups based on culture conditions: K562R+ima, maintained in medium containing 5 μM IMA, and K562R−ima, cultured without IMA. This subdivision was carried out to evaluate the effect of E7820 both in the presence and absence of IMA, allowing assessment of its efficacy as a single-agent therapy.

### 2.2. Cell Viability Assay

K562S and K562R cells (4 × 10^4^ cells/well) were harvested into 96-well plates and incubated with E7820 (ITGA2 inhibitor) (Sigma, St. Louis, MA, USA). K562S cells were treated with E7820 alone to evaluate its effect on cell viability. In contrast, K562R cells were treated with E7820 both as a single agent and in combination with IMA. Cell proliferation was assessed using the MTT assay after 72 h of treatment with increasing concentrations of E7820 (0.06, 0.125, 0.25, 0.5, 1, 2.5, 5, 7.5, 10, 15, 20, and 23 μM). Absorbance was measured at 550 nm with 690 nm as a reference using a spectrophotometric plate reader (Biotek, Winooski, VT, USA). Cell viability in untreated controls was set as 100%, and the viability of E7820-treated group was determined relative to this baseline. DMSO, used to dissolve E7820, functioned as the negative control, and its concentration was kept consistent across all treatment conditions.

### 2.3. Analysis of ITGA2 Protein Expression

Flow cytometry analysis was conducted to evaluate the inhibitory effect of E7820 (2.5 μM) on ITGA2 protein expression. K562S and K562R cells (5 × 10^5^ cells/well) were seeded into 24-well plates and treated with E7820 at concentrations of 2.5 µM for 72 h. To assess ITGA2 inhibition by E7820, all cell groups were treated with E7820. Later, cells were washed with PBS and incubated with a primary anti-human ITGA2 antibody (Invitrogen, Carlsbad, CA, USA, Thermo Fisher Scientific/Waltham, MA, USA) for 30 min at 4 °C. Following a wash, cells were stained with a secondary antibody, Alexa Fluor^®^ 488, (Invitrogen, Carlsbad, CA, USA, Thermo Fisher Scientific, Waltham, MA, USA) for 30 min at 4 °C. Samples were analyzed on a flow cytometer (Accuri™ C6, BD Biosciences, San Jose, CA, USA), and data were expressed as mean fluorescence intensity (MFI).

### 2.4. Flow Cytometry for Annexin V

K562S and K562R cells (5 × 10^5^ cells/well) were plated in 24-well plates and exposed to 2.5 µM E7820 for 72 h. Following incubation, cells were centrifuged and resuspended in 1× binding buffer (BB). For apoptosis analysis, 100 μL of the cell suspension was stained with PE-Annexin V and 5 μL 7-AAD (5 μL) (BD Pharmingen™ PE Annexin V Apoptosis Detection Kit I) (BD Biosciences, San Jose, CA, USA), then incubated at room temperature for 15 min. After staining, 400 μL of 1× BB was added, and samples were analyzed for apoptosis using flow cytometry (Accuri™ C6, BD Biosciences, San Jose, CA, USA).

### 2.5. Caspase 3/7 Activity

K562S and K562R cells (5 × 10^5^ cells/well) were cultivated into 24-well plates treated with 2.5 μM E7820 for 72 h. Caspase-3/7 enzymatic activity was assessed using the CellEvent™ Caspase-3/7 Green Detection Reagent (Invitrogen, Carlsbad, CA, USA, Thermo Fisher Scientific, Waltham, MA, USA) in line with the protocol supplied by the manufacturer. Then, cells were incubated with 1 μL of the detection reagent for 30 min at 37 °C. Prior to flow cytometry analysis (Accuri™ C6, BD Biosciences, San Jose, CA, USA), 1 μL of 1 mM SYTOX™ AADvanced™ Dead Cell Stain Solution was added to the cell suspension. Apoptotic and dead cell populations were quantified based on caspase-3/7 activation and membrane permeability by measuring MFI.

### 2.6. Real-Time Quantitative RT-qPCR

To assess the expression of apoptosis-related genes (*BAX*, *BIM*, *BAD*, and *BCL-2*), K562S, K562R-ima, and K562R+ima cells (4 × 10^5^ cells/well) were cultured in 24-well plates and incubated with 2.5 μM E7820 for 72 h. Total RNA was extracted using TRIzol reagent (Invitrogen, Carlsbad, CA, USA), and cDNA was generated with the iScript cDNA Synthesis Kit (Bio-Rad, Hercules, CA, USA) following the manufacturer’s protocol. Quantitative real-time PCR was carried out using iTaq Universal SYBR Green Supermix on a CFX Connect System (Bio-Rad, Hercules, CA, USA). In our study, *HPRT* (hypoxanthine-guanine phosphoribosyltransferase) was selected as the housekeeping gene for qRT-PCR normalization due to its stable expression across the experimental conditions and cell lines used (K562S and K562R). Gene expression levels were normalized to *HPRT*, and the primers used are detailed in Table 1. In this study, we analyzed relative mRNA expression levels using the 2^−ΔCt^ method, normalizing target gene expression to the housekeeping gene *HPRT*.

### 2.7. P-Glycoprotein Activity Measurement in Cell Lines

Assay was performed to assess P-glycoprotein (P-gp) activity, based on its ability to actively transport Rhodamin 123 (Rho-123) dye out of cells. K562S and K562R cells (5 × 10^5^ cells/well) were seeded in 24-well plates and treated with 2.5 μM of E7820 for 72 h. After treatment, cells were incubated with Rho-123 (150 ng/mL; Sigma, St. Louis, MA, USA) in RPMI 1640 medium at 37 °C for 30 min. Cells were then washed twice with PBS, resuspended in fresh RPMI 1640 medium, and allowed to undergo dye efflux for 90 min at 37 °C. Following this, cells were washed again with PBS and analyzed by flow cytometry (Accuri™ C6, BD Biosciences, San Jose, CA, USA) to quantify intracellular Rho-123 retention, which inversely reflects P-gp activity.

### 2.8. Bioinformatic Analysis

To support and validate our experimental findings obtained from CML cell lines, we incorporated a bioinformatics approach using publicly available gene expression datasets. Gene expression data were obtained from the GSE47927 (https://www.ncbi.nlm.nih.gov/geo/query/acc.cgi?acc=GSE47927, accessed on 19 August 2025) and GSE51082 (https://www.ncbi.nlm.nih.gov/geo/query/acc.cgi?acc=GSE51082, accessed on 19 August 2025) series available in the Gene Expression Omnibus (GEO) database. Clinical and experimental groupings of the samples were identified using the metadata provided in the GEO dataset (https://www.ncbi.nlm.nih.gov/geo/ accessed on 19 August 2025). Probes corresponding to the *ITGA2* gene were selected using the platform annotation file (GPL), and normalized; log2-transformed expression values were extracted from the Series Matrix file. The resulting *ITGA2* expression values were imported into GraphPad Prism (v10.x) in a two-column format grouped by condition. Following normality testing, statistical analysis was performed using T test and post hoc Mann–Whitney U test for comparisons between two groups. A *p*-value < 0.05 was considered statistically significant.

To assess *ITGA2* expression levels in clinical samples from CML patients, we performed bioinformatics analysis with the study numbered GSE47927 series and GPL6244 platform. Relevant datasets containing transcriptomic profiles of CD34+ samples from CML patient were selected to compare *ITGA2* expression across various disease stages, including chronic phase (CP), accelerated phase (AP), and blast crisis (BC).

Additionally, we used the GSE51082 dataset (also based on the GPL96 and GPL97 platforms) to analyze mononuclear cell samples from patients with various hematologic malignancies, including acute myeloid leukemia (AML), B-cell acute lymphoblastic leukemia (B-ALL), T-cell acute lymphoblastic leukemia (T-ALL), chronic lymphocytic leukemia (CLL), CML, and myelodysplastic syndromes (MDSs). The aim was to characterize the *ITGA2* expression profile across different leukemia subtypes, providing a broader context for its potential role in leukemogenesis.

### 2.9. Statistical Analysis

Version 10 of GraphPad Prism (GraphPad Software, San Diego, CA, USA) was used to conduct the statistical analysis. Significant differences between groups in the MTT cell viability assay, apoptosis, and RT-PCR assays were identified using One Way Anova and Two Way Anova with Tukey’s multiple comparisons testing, and *t*-Test, respectively. The standard deviation, or mean ± SD, was applied to all experimental data. At *p* < 0.05, *p* < 0.01, *p* < 0.001, and *p* < 0.0001, statistical significance was established.

## 3. Results

### 3.1. Cytotoxic Effects of E7820 on CML Cell Lines

**Figure 1:**
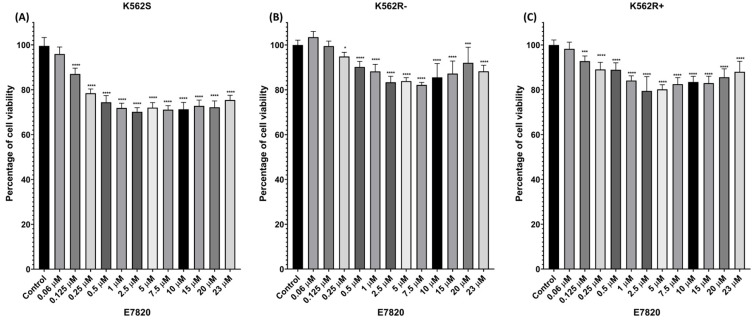
Cells were treated with increasing concentrations of E7820 (0.06, 0.125, 0.25, 0.5, 1, 2.5, 5, 7.5, 10, 15, 20, and 23 μM), and cell viability was assessed. (**A**) K562S, (**B**) K562R-ima, and (**C**) K562R+ima cells were treated with E7820 and compared to untreated controls. (*n* = 4) (* *p* < 0.05, *** *p* < 0.001, **** *p* < 0.0001).

To evaluate the cytotoxic effect of E7820, K562S, K562R-ima, and K562R+ima cells were treated with increasing concentrations of the drug (0.06, 0.125, 0.25, 0.5, 1, 2.5, 5, 7.5, 10, 15, 20, and 23 μM) for 72 h, and cell viability was assessed using the MTT assay. Viability was normalized to DMSO-treated control cells (set at 100%). As shown in Figure 1A–C, E7820 induced a dose-dependent reduction in cell viability in all three cell lines between 0.06 and 2.5 μM doses. A notable and statistically significant reduction in viability was observed starting at 2.5 μM in all CML cells (*p* < 0.0001). The results showed that 2.5 µM was the lowest tested concentration that led to a marked reduction in cell viability across all cell lines, relative to the untreated controls, and that no potent decrease in viability values was observed at higher doses. Therefore, 2.5 µM was selected for use in subsequent experiments.

### 3.2. Effect of E7820 on ITRGA2 Protein Level in CML Cells

**Figure 2:**
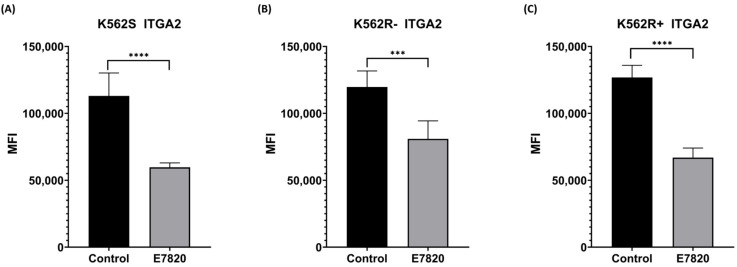
Protein levels of ITGA2 were analyzed by flow cytometry. Columns indicate mean fluorescence intensity in (**A**) K562S; (**B**) K562R-ima; (**C**) K562R+ima. (*n* = 4) (*** *p* < 0.001 and **** *p* < 0.0001).

To analyze the inhibitory effect of E7820 (2.5 μM) on ITGA2 protein levels, we carried out flow cytometry analysis. According to the obtained data, ITGA2 protein levels were decreased significantly in all E7820-treated K562S and K562R-ima and K562R+ima cells compared to the control cells (Figure 2A–C). In K562R cells, treatment with E7820 in combination with IMA led to a more substantial decrease in ITGA2 protein levels than treatment with IMA alone, suggesting enhanced efficacy of the combination approach.

### 3.3. Effect of E7820 in Apoptosis Induction of CML Cells

To evaluate the mode of cell death induced by E7820 at concentration of 2.5 μM for 72 h, Annexin V/7-AAD staining was used to distinguish between viable, early apoptotic, late apoptotic, and necrotic cells. In this assay, Annexin V^−^/7-AAD^−^ cells are considered viable, Annexin V^+^/7-AAD^−^ represent early apoptotic cells, Annexin V^+^/7-AAD^+^ are late apoptotic or dead cells, and Annexin V^−^/7-AAD^+^ indicate necrotic cells.

**Figure 3:**
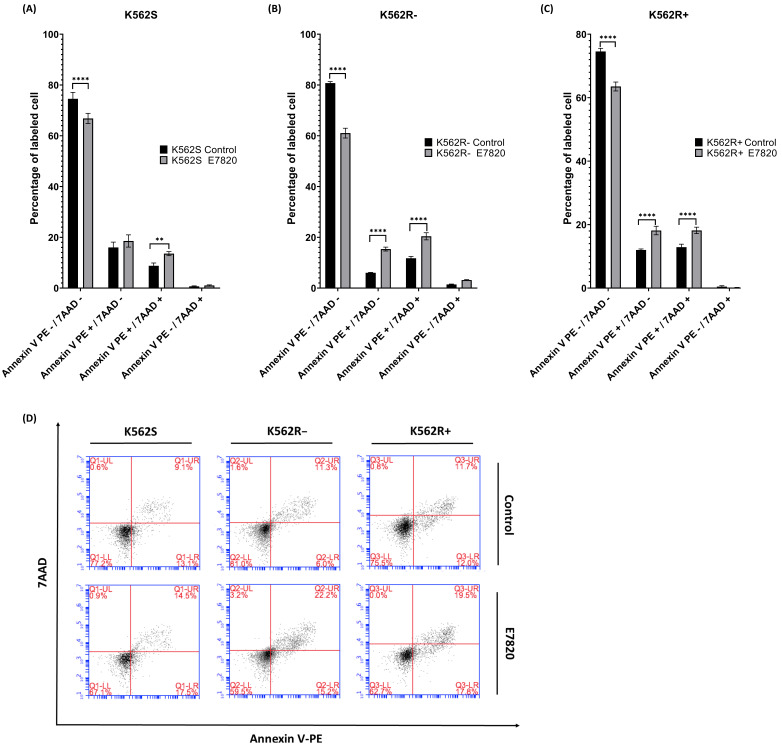
Flow cytometric analysis using Annexin V-PE/7-AAD-stained K562S and K562R cells. Quantification of apoptotic populations (bar graph) in (**A**) K562S; (**B**) K562R-ima; (**C**) K562R+ima cells; (**D**) Dot plot representation of flow cytometry data. (*n* = 4) (** *p* < 0.01, and **** *p* < 0.0001).

K562S cells incubated with 2.5 μM E7820 for 72 h demonstrated a significant shift in cell populations compared to untreated controls. Specifically, there was an increase in both early apoptotic (Annexin V^+^/7-AAD^−^) and late apoptotic/dead cells (Annexin V^+^/7-AAD^+^) (*p* < 0.01), along with a significant reduction in viable cells (Annexin V^−^/7-AAD^−^) (*p* < 0.0001) (Figure 3A), indicating that E7820 effectively induces apoptosis in IMA-sensitive cells.

In K562R-ima cells, treatment with E7820 alone (2.5 μM) similarly resulted in a significant increase in both early and late apoptotic populations (*p* < 0.0001), along with a notable decrease in viable cells (*p* < 0.0001) (Figure 3B). For K562R+ima cells, the combined treatment of E7820 and 5 μM IMA led to the high levels of apoptosis significantly increasing both early and late apoptotic cells compared to IMA-only treatment (*p* < 0.0001) (Figure 3C). The percentage of viable cells was also substantially reduced (*p* < 0.0001), indicating an effect of the combination therapy in overcoming resistance and promoting cell death. These findings demonstrate that E7820, particularly when used in combination with IMA, effectively induces apoptosis in both sensitive and resistant CML cell lines, highlighting its potential as a therapeutic strategy to increase therapy efficacy in IMA-resistant cases.

### 3.4. Effect of E7820 on Caspase 3/7 Activation in CML Cells

**Figure 4:**
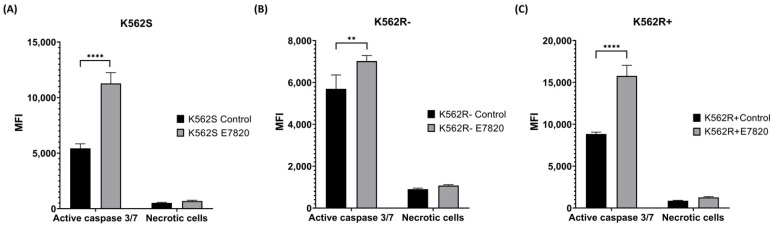
Effects of E7820 (2.5 μM) on caspase3/7 activity in treated K562S and K562R cells compared to their untreated counterparts. (**A**) K562S; (**B**) K562R-ima; (**C**) K562R+ima cells (*n* = 4) (** *p* < 0.01 and **** *p* < 0.0001).

To assess caspase-mediated apoptosis, caspase-3/7 enzymatic activity was measured, as these enzymes serve as key indicators of caspase-dependent apoptotic pathways. E7820 incubation at 2.5 μM for 72 h significantly elevated caspase-3/7 activity in both K562S and K562R-ima cells compared to untreated controls (*p* < 0.0001 and *p* < 0.01) (Figure 4A, B). Moreover, the combination of E7820 with 5 μM IMA significantly elevated caspase-3/7 activity in K562R+ima cells relative to IMA-only treatment, indicating a more potent apoptotic response (*p* < 0.0001) (Figure 4C). All these results suggest the involvement of caspases 3 and 7 in E7820-induced apoptosis.

### 3.5. Effect of E7820 on Expression Profiling of Apoptotic Genes in CML Cells

**Figure 5:**
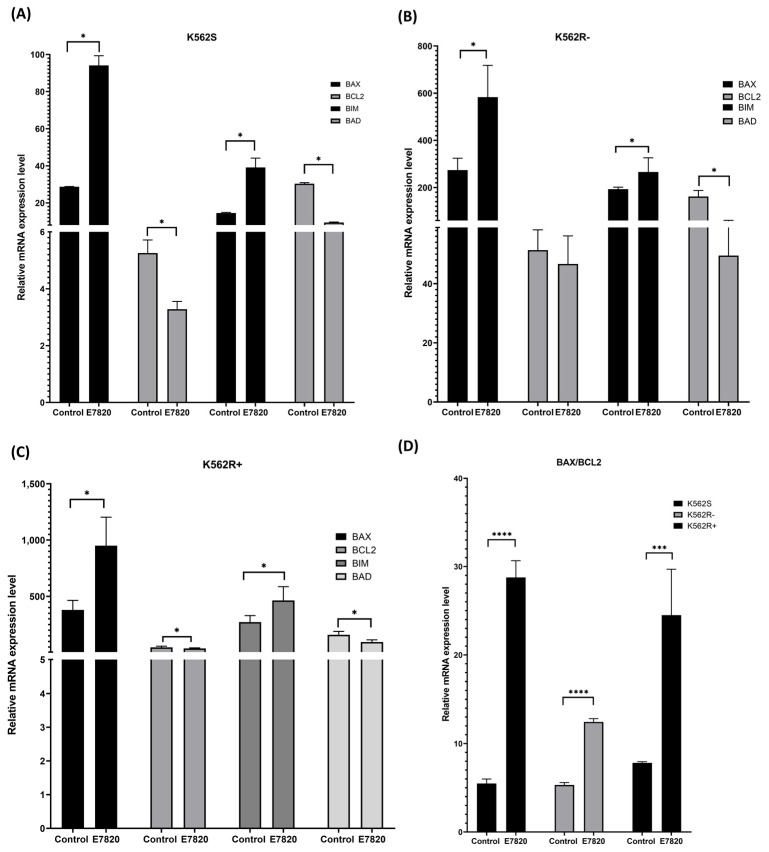
Gene expression analysis of K562S and K562R cells upon incubation with E7820 for 72 h by real-time PCR assay. Effects of E7820 treatment *BAX*, *BAD*, *BIM*, and *BCL-2* gene expressions; (**A**) K562S; (**B**) K562R-ima; (**C**) K562R+ima cell; (**D**) Bar graph presentation of *BAX/BCL-2* mRNA ratio. (*n* = 4) (* *p* < 0.05, *** *p* < 0.001, **** *p* < 0.0001).

The impact of E7820 on the mRNA expression of *BCL-2* family genes (*BAX*, *BIM*, *BAD*, and *BCL-2*) was evaluated using qRT-PCR. In K562S cells, E7820 treatment significantly upregulated *BAX* and *BIM* (pro-apoptotic genes) and downregulated the *BCL-2* (anti-apoptotic gene) (*p* < 0.05) (Figure 5A).

E7820 treatment (2.5 μM) in K562R-ima cells enhanced the expression of both *BAX* and *BIM* significantly (*p* < 0.05) and decreased *BCL-2* genes in comparison with the untreated controls (Figure 5B). Combination treatment of E7820 with 5 μM IMA in K562R+ima cells led to an upregulation of *BAX* and *BIM* genes significantly (*p* < 0.05), and downregulation of *BCL-2* gene-treated cells (*p* < 0.05) (Figure 5C).

Interestingly, *BAD* expression was significantly decreased in E7820-treated K562S, K562R-ima, and K562R+ima cells (*p*< 0.05) (Figure 5A).

### 3.6. Effect of E7820 on Intracellular Accumulation of Rho-123 in CML Cells

**Figure 6:**
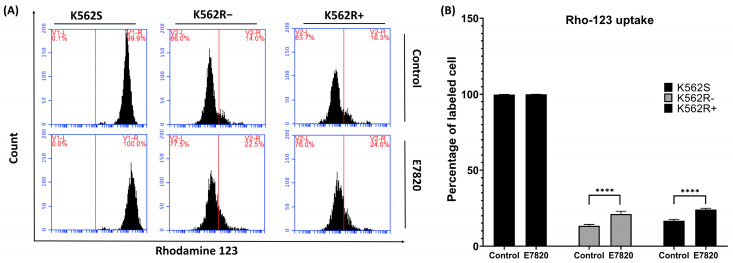
Intracellular accumulation of Rho-123 was assessed by flow cytometry in K562S, K562R-ima, and K562R+ima cells. (**A**) Flow cytometry results are represented as dot; (**B**) Bar graph representation of flow cytometry data. (*n* = 4) (**** *p* < 0.0001).

E7820 treatment (2.5 μM) did not change Rho-123 uptake in K562S cells compared to untreated controls. In K562R-ima cells, MFI of Rho-123 uptake was dramatically elevated at 2.5 μM E7820 in treated cells compared to the cells without inhibitor (*p* < 0.0001). Similarly, E7820 treatment (2.5 μM) significantly enhanced Rho-123 uptake in K562R+ima cells compared to the control (*p* < 0.0001) (Figure 6A, B).

### 3.7. Differential ITGA2 Expression Across CML Stages and Leukemia Subtypes

**Figure 7:**
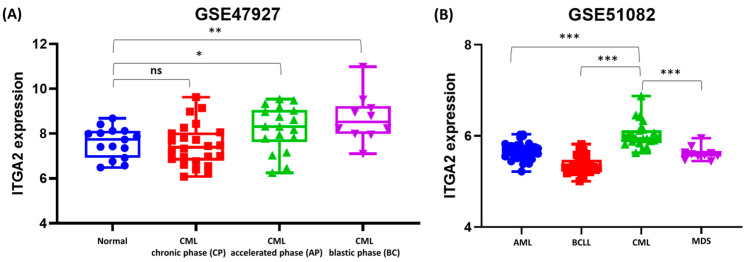
*ITGA2* gene expression in CML disease stages and leukemia subtypes. (**A**) *ITGA2* expression in CD34+ cells from healthy controls and CML patients in CP, AP, and BC based on the GSE47927 dataset; (**B**) *ITGA2* expression in mononuclear cells from patients with AML, B-ALL, T-ALL, B-CLL, CML, and MDS. Data were obtained from the GEO dataset GSE47927 and GSE51082 (ns: not significant, * *p* < 0.05, ** *p* < 0.01, and *** *p* < 0.001).

We assessed *ITGA2* gene expression in CD34+ hematopoietic stem/progenitor cells obtained from CML patients at different disease stages (CP, AP, and BC) and compared to the healthy volunteers. The analysis showed that *ITGA2* gene expression was significantly upregulated in CML patients, with the highest levels observed in the AP (*p* < 0.05) and BC (*p* < 0.01) phases compared to the healthy group (Figure 7A).

Furthermore, we conducted bioinformatic analyses on mononuclear cells from BM and peripheral blood of leukemia patient samples (AML, B-ALL, T-ALL, B-CLL, CML, and MDS) datasets. The results showed that *ITGA2* expression significantly varied across these leukemia types. Notably, CML exhibited elevated *ITGA2* levels compared to the AML, B-CLL, and MDS (*p* < 0.001) (Figure 7B). These findings suggest a positive correlation between *ITGA2* expression and disease progression, indicating that *ITGA2* may play a role in leukemic transformation.

## 4. Discussion

Integrins constitute a large family of molecules that play a key role in the regulation of multicellular biological processes. They coordinate cell-cell and cell–ECM adhesion, from embryonic development through to the maintenance of mature tissue function [19]. Aberrant integrin-mediated adhesion is implicated in a wide range of human diseases, including thrombosis, inflammation, cancer, fibrosis, and infectious diseases [9].

It has also been shown that integrins contribute to chemotherapy resistance. Their accessibility on the cell surface and responsiveness to pharmacological inhibition make them promising therapeutic targets [19]. The association of integrins and ECM with hematological malignancy development was reported in various studies [20,21].

In our previous study, we found *ITGA2* is overexpressed in K562R cells compared to the K562S cells [6]. In parallel with all these studies, its inhibition in these cells, specially in combination with IMA, induced apoptosis. To explore the role of *ITGA2* in CML and IMA resistance, we used ITGA2 inhibitor (E7820) to suppress this cell surface molecule.

To disclose the mechanisms underlying apoptosis induced by E7820, we performed MTT, annexin V, and caspase 3/7 flow cytometry assays. According to the results, we showed that 2.5 µM of E7820 decreased cell viability significantly in all cells. It also induced apoptosis in all K562S, K562R-ima, and K562R+ ima cells. The percentage of apoptotic cells in the cell groups which were treated with both inhibitor and IMA together is higher than the cells treated with inhibitor. To further investigate apoptosis induced by E7820, we analyzed the expression levels of pro-apoptotic (*BAX*, *BIM*, and *BAD*) and anti-apoptotic (*BCL-2*) genes. Based on the obtained data, single or combination treatment of inhibitor increased the mRNA expression levels of *BAX* and *BIM* genes and decreased the *BCL-2* gene expression in all cell groups whereas *BAD* gene expressions were reduced in all treated groups. Our data demonstrated that E7820 induced apoptosis through modulation of *BAX* and *BCL*-2 and a high *BAX*/*BCL-2* ratio was achieved in all cells after 2.5 µM E7820 treatment.

In parallel with our findings, several studies have highlighted the role of integrins, particularly *ITGA2*, in cancer progression, and have also demonstrated that integrin inhibition may enhance therapeutic efficacy. In the study carried out by Hsieh and colleagues, it was shown that *ITGA4* plays a key role in protecting ALL cells from chemotherapy by mediating their adhesion to bone marrow stromal cells. Disrupting alpha4 either genetically or with the antibody Natalizumab sensitized leukemia cells to treatment and improved survival in preclinical models. These findings suggest that targeting *ITGA4* could be a promising adjuvant strategy to enhance chemotherapy effectiveness in pre-B ALL [22]. The role of *ITGA7* and ECM in AML with granulocytic sarcoma (GS) cells was first reported in the study performed by Kobayashi and colleagues [20]. CD49d (α4:β1) has been shown to predict overall survival in CLL and is involved in chemo-resistance in ALL and AML; in CLL, it contributes to fludarabine resistance by inducing *Bcl-xL* overexpression and inhibiting apoptosis [21,23,24,25].

Recent studies have increasingly reported abnormal ITGA2 expression across a range of malignancies, including gastric, pancreatic, breast, liver cancers, and glioma, where it plays a crucial role in various aspects of tumor initiation and progression [26]. It was reported that silencing of *ITGA2* strengthened TGF-β’s ability to suppress pancreatic cancer cell proliferation and tumor growth [27]. Moreover, integrin α2/β1 promoted peritoneal dissemination of gastric cancer by increasing cell adhesion and metastasis, and its inhibition significantly reduced tumor spread, making it a potential therapeutic target [28]. Analysis of primary and metastatic colorectal cancer samples demonstrated that *ITGA2* promotes liver metastasis, identifying it as a potential target for preventing hepatic spread and metastasis [18]

The investigation conducted by Korovina et al. reported that *ITGA2* is upregulated in glioblastoma (GBM), and its deletion elevates radio-chemosensitivity and decreases invasion, impairing proliferation [29]. Moreover, another study showed that *ITGA2* is upregulated in intrahepatic cholangiocarcinoma (iCCA) and promotes tumor growth by mediating strong interactions with collagen type I in the extracellular matrix, suggesting the collagen type I–integrin α2 axis as a potential therapeutic target for iCCA [30].

In addition to the role of *ITGA2* in cancer progression, there are studies demonstrating its role in mediating resistance. Chemotherapy resistance contributes to poor outcomes in gastric cancer (GC), and overexpressed *ITGA2* has been observed in chemotherapy-resistant cells, suggesting a role in mediating resistance. It is reported that *ITGA2* is upregulated in chemo-resistant gastric cancer (GC) cells and correlates with poor prognosis, while its knockdown restores chemo-sensitivity by downregulating MAPK/ERK signaling and EMT; additionally, miR-135b-5p directly targets ITGA2, reducing chemo-resistance and highlighting the miR-135b-5p/ITGA2 axis as a potential diagnostic and therapeutic target in GC [31]. Dong et al. identified a novel HMGA2-FOXL2-ITGA2 signaling pathway that contributes to chemotherapy resistance and metastasis in GC [32].

Anoikis resistance, essential for cancer metastasis, is promoted in cancer stem cells (CSCs) by elevated *ITGA2* and *ITGB1* expression, which interact with EGFR to activate the ERK/Akt survival pathway, enabling CSCs to evade cell death in the absence of extracellular matrix [33].

A recent study has demonstrated that loss of *ITGA2* reduces glioma cell proliferation, invasion, and adhesion, and can be targeted to overcome radio- and chemo-resistance, ultimately improving patient survival [29]. High *ITGA2* expression is associated with lower complete remission rates and shorter overall survival in AML patients, with levels dropping in remission and rising at relapse, indicating its role as a poor prognostic marker; additionally, α2β1-mediated adhesion to collagen I in T-ALL cells reduces doxorubicin-induced apoptosis, contributing to chemo-resistance [34]. α2β1-mediated adhesion activates the MAPK/ERK pathway, which suppresses doxorubicin-induced JNK activation and sustains the pro-survival protein Mcl-1, contributing to drug resistance; this mechanism was also observed in AML cell lines HL-60 and U937, further supporting α2β1-collagen’s role in chemo-resistance [35].

In our study, we used E7820, an integrin inhibitor, and this compound has also been reported in previous studies to play a therapeutic role in cancer by targeting integrin-mediated pathways. E7820 is an angiogenesis inhibitor that reduces *ITGA2* expression and is currently undergoing clinical trials [36]. Matsumotu et al. examined *ITGA2* expression in 100 non-small-cell lung cancer patients and found that high expression was associated with lower recurrence-free survival. In vitro experiments represented that overexpression of *ITGA2* enhanced cell size, adhesion, and migration but did not affect proliferation or invasion. These effects were inhibited by E7820, suggesting *ITGA2* may contribute to cancer recurrence through elevated adhesion and migration [37]. In another study, the role of *ITGA2* in methotrexate (MTX)-induced epithelial-mesenchymal transition (EMT) in lung epithelial cells was investigated. MTX significantly elevated *ITGA2* expression, which correlated with enhanced EMT marker expression, while co-treatment with EMT inhibitors suppressed this effect. The ITGA2 inhibitor E7820 also reduced MTX-induced EMT changes, suggesting *ITGA2* plays a critical role in mediating MTX-induced lung epithelial injury [38].

It has been reported that incubation of K562 cells with E7820 (1 µM) for 24 h resulted in the selective degradation of CAPERα, demonstrating the compound’s potential to disrupt RNA splicing and affect cancer cell viability through targeted splicing factor modulation [39].

In our current study, the observed relationship between *ITGA2* expression and MDR-1(P-gp) activity is correlative. We found that MDR activity decreased in all inhibitor-treated K562R cells by performing the rhodamine assay. P-gp encoded by the *ABCB1* gene is an ATP-dependent efflux pump that transports a broad range of substances, including chemotherapeutic agents, immunosuppressants, and steroids. Its overexpression in various cancers has been linked to reduced chemotherapy effectiveness and poor clinical outcomes [40].

Several studies have shown that integrins can upregulate P-gp, a key drug efflux transporter, thereby contributing to chemotherapy resistance in various cancers. For instance, αvβ6 and β1 integrins enhance P-gp expression in doxorubicin-resistant breast cancer cells (MCF-7/ADR) and cisplatin-resistant laryngeal cancer cells, respectively [41,42]. Similarly, the interaction between αvβ3 integrin and osteopontin increases P-gp expression in prostate cancer cells (PC-3), leading to resistance against multiple chemotherapeutic agents [43]. These findings suggest that targeting integrin-mediated regulation of drug transporters may be a promising strategy for overcoming drug resistance in cancer therapy. In our previous study, we demonstrated that MDR-1 expression was upregulated in K562R cells compared to K562S cells, which is consistent with reports of increased P-glycoprotein (P-gp) activity in K562R cells relative to K562S cells [5]. Whereas in the current study we showed that E7820 treatment decreased the MDR activity and led to accumulation of the E7820 in the cell. Collectively, these results indicate that ITGA2 inhibition in all treated cells induces apoptosis by reducing MDR activity, increasing intracellular E7820 levels, and modulating apoptotic gene expression and caspase-3/7 activity.

Although we were unable to include primary CML patient samples due to experimental limitations, we performed bioinformatic analyses using publicly available datasets from patients to support and extend our in vitro findings. According to our data, *ITGA2* expression is significantly higher in CML patients in accelerated and blast phases compared to the normal volunteers. In addition, data represented that *ITGA2* expression changes across leukemia types. Notably, CML exhibited significantly increased *ITGA2* levels compared to the AML, B-CLL, and MDS.

We previously reported that *ITGA2* is upregulated in K562R cells compared to K562S cells [6], and this finding is further supported by bioinformatic analysis of CML patient datasets, suggesting a potential role for ITGA2 in drug resistance and clinical relevance. A recent Phase II clinical trial demonstrated that the anti-cancer sulfonamide E7820 effectively degrades RBM39 in patients with relapsed or refractory myeloid malignancies, including AML, MDS, and chronic myelomonocytic leukemia (CMML). The treatment showed an acceptable safety profile, with common side effects such as diarrhea, anemia, neutropenia, and fatigue; however, clinical responses were limited when used as a single agent [44]. Although specific ITGA2 inhibitors are not yet clinically available, future studies should investigate whether ITGA2 inhibition could synergize with TKIs to improve treatment outcomes and overcome resistance in CML patients.

## 5. Conclusions

This study is the first to investigate the role of ITGA2 in chronic myeloid leukemia (CML), with a particular focus on imatinib (IMA)-resistant CML. While ITGA2 has been extensively studied in various solid tumors and other hematologic malignancies, including its involvement in cell adhesion, metastasis, and drug resistance, its contribution to CML pathogenesis and therapy resistance remained unexplored until now. Our findings provide novel evidence that ITGA2 may play a functional role in the development of resistance to imatinib, a frontline tyrosine kinase inhibitor used in CML treatment. These results suggest that ITGA2 could serve as a potential biomarker for therapy resistance and may represent a promising therapeutic target for overcoming drug resistance in CML. In conclusion, while our findings suggest a potential role for ITGA2 in drug resistance, they remain preliminary and require further validation in primary patient samples and in vivo models.

## Figures and Tables

**Table 1:** Primer details for real-time qRT-PCR.

Gene	Forward (5′-3′)	Reverse (5′-3′)
*BAX*	GACGCAACTTCAACTGGG	AGGAGTCTCACCCAA CAC
*BIM*	ATCTCACAATGGCTTCC	CATAGTAAGCGTTAAACTCGTCTCC
*BAD*	GATGAGTGACGAGTTTGTGGA	CAAGTTCCGATCCCACCAG
*BCL2*	CGCCCTGTGGATGACTGAGT	GGGCCGTACAGTTCCACAA
*HPRT*	TGACACTGCAAAACAATGCA	GGTCCTTTTCACCAGCAAGCT

## Data Availability

The original contributions presented in this study are included in the article. Further inquiries can be directed to the corresponding authors.

## Abbreviations

CML	chronic myeloid leukemia
IMA	imatinib
ITGA2	integrin A2
K562S	IMA-sensitive K562
K562R	IMA-resistant K562
µM	micromolar
ECM	extracellular matrix
SNPs	single nucleotide polymorphisms
ALL	acute lymphoblastic leukemia
AML	acute myeloid leukemia
B-ALL	B-cell acute lymphoblastic leukemia
T-ALL	T-cell acute lymphoblastic leukemia
CLL	chronic lymphocytic leukemia
B-CLL	B-cell chronic lymphocytic leukemia
CMML	chronic myelomonocytic leukemia
MDS	myelodysplastic syndromes
P-gp	P-glycoprotein
FBS	fetal bovine serum
MFI	mean fluorescence intensity
BB	binding buffer
Rho-123	Rhodamin 123
CP	chronic phase
AP	accelerated phase
BC	blast crisis
GEO	Gene Expression Omnibus
